# When Lupus Isn’t Lupus: Erythema Marginatum in an Adult with a History of Acute Rheumatic Fever

**DOI:** 10.7759/cureus.111702

**Published:** 2026-06-29

**Authors:** Chaimae Bouhamdi, Zakia Douhi, Kawtar El Fid, Hanane Baybay, Fatima Zahra Mernissi

**Affiliations:** 1 Department of Dermatology, University Hospital Hassan II, Fez, MAR

**Keywords:** acute rheumatic fever, annular erythema, erythema marginatum, rheumatic carditis, subacute cutaneous lupus erythematosus

## Abstract

Erythema marginatum is a rare but diagnostically valuable cutaneous manifestation of acute rheumatic fever (ARF), particularly in young individuals with a history of streptococcal infection. Its presentation is often transient and underrecognized in adult populations, especially when mimicking other annular dermatoses such as subacute cutaneous lupus erythematosus (SCLE). A young woman in her early twenties with a history of ARF and residual rheumatic valvular involvement presented with recurrent, asymptomatic annular erythematous plaques. Initial diagnostic considerations included dermatophytosis and SCLE. Skin biopsy showed chronic dermal inflammation with a perivascular and periadnexal lymphoplasmacytic infiltrate, without lupus-specific histopathologic features. In the appropriate rheumatic context, these findings supported erythema marginatum as a clinicopathologic diagnosis of exclusion.

Our case highlights the importance of considering erythema marginatum in the differential diagnosis of recurrent annular dermatoses in patients with a history of ARF. Clinicopathologic correlation remains essential to ensure appropriate management.

## Introduction

Erythema marginatum is a rare cutaneous manifestation of acute rheumatic fever (ARF), classified among the major Jones diagnostic criteria [1,2]. Pathogenetically, ARF is an immune-mediated sequela of group A Streptococcus infection, in which cross-reactive host immune responses through molecular mimicry may lead to inflammatory involvement of the heart, joints, skin, and central nervous system [1]. It typically presents as evanescent, annular, non-pruritic erythematous plaques with central clearing, most often observed on the trunk and proximal extremities [2,3]. Although classically described in children and adolescents, it is rarely reported in adults, in whom recognition may be delayed, particularly when lesions occur outside a classic acute febrile episode or follow a recurrent course [4-6]. The diagnostic difficulty is further compounded by the lesion’s clinical overlap with several annular dermatoses, such as tinea corporis, subacute cutaneous lupus erythematosus (SCLE), and erythema annulare centrifugum [7,8]. Lacking pathognomonic histopathologic features, erythema marginatum remains a clinicopathologic diagnosis of exclusion, requiring correlation between morphology, rheumatic history, and exclusion of mimickers [2,8-10]. Recognition is clinically significant, as it may point to an underlying rheumatic context or recurrent rheumatic activity, with implications for cardiac surveillance and continuation of secondary prophylaxis [2,11].

We report an adult case of recurrent erythema marginatum in a patient with a history of ARF and residual rheumatic valvular involvement. The eruption was initially misinterpreted as dermatophytosis and SCLE, and the diagnosis was ultimately supported by integration of the clinical morphology, histopathologic findings, and the patient’s rheumatic history.

## Case presentation

**Figure 1:**
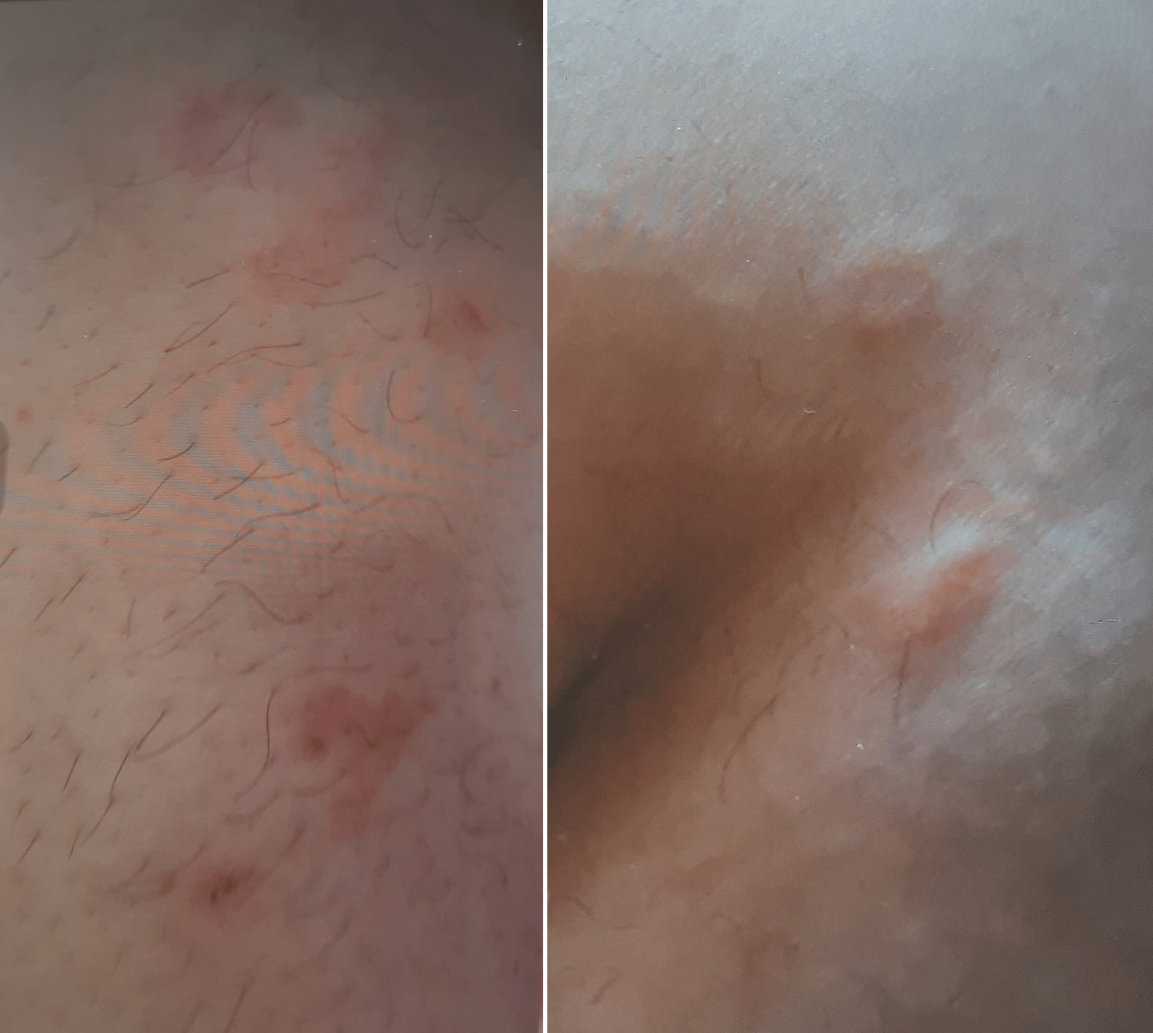
Patient-provided clinical photographs from a prior flare, showing multiple oval erythematous plaques with infiltrated borders.

A young woman in her early twenties, with a history of acute rheumatic fever (ARF) diagnosed in early adolescence, and complicated by rheumatic carditis with residual thickening and remodeling of the mitral valve, under secondary prophylaxis with benzathine benzylpenicillin, was referred by internal medicine for dermatologic evaluation of suspected subacute cutaneous lupus erythematosus (SCLE). She reported a two-year history of recurrent, transient, asymptomatic erythematous plaques with minimal scaling and a relapsing-remitting course, characterized by fading of previous lesions and the appearance of new plaques at different sites. Three months earlier, a flare had been diagnosed as dermatophytosis and treated with topical terbinafine, with apparent resolution. Patient-provided clinical photographs showed multiple oval erythematous plaques with infiltrated borders (Figure 1).

**Figure 2:**
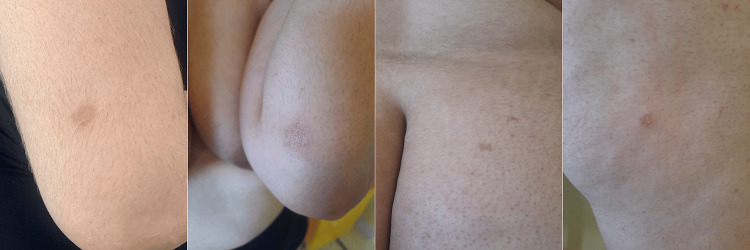
Oval erythemato-pigmented plaques on the forearms and well-demarcated erythematous plaques on the left thigh with slightly more infiltrated borders.

One week before the consultation, she developed non-pruritic, non-painful erythematous oval macules and plaques on the forearms and lower limbs. Systemic symptoms included inflammatory polyarthralgia. Examination revealed five oval erythemato-pigmented plaques on the forearms and two well-demarcated erythematous plaques on the left thigh with slightly more infiltrated borders (Figure 2).

**Figure 3:**
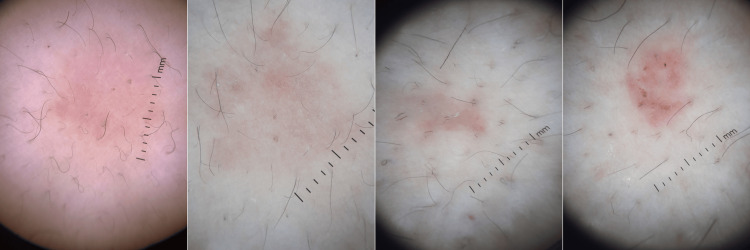
Corresponding dermoscopy of the same clinical lesions, showing a faint pink background with sparse dotted vessels in a nonspecific inflammatory pattern.

Dermoscopy revealed a faint pink background with sparse dotted vessels, consistent with a nonspecific inflammatory pattern (Figure 3).

Initial differential diagnosis included SCLE, eczema, and dermatophytosis.

Laboratory testing showed a complete blood count and renal function within reference ranges. The erythrocyte sedimentation rate was normal, whereas C-reactive protein was mildly elevated. Immunologic testing revealed weakly positive anti-dsDNA antibodies, with negative ANA, anti-SSA/Ro antibodies, and anti-SSB/La antibodies (Table 1).

**Table 1:** Laboratory and immunologic findings at dermatologic evaluation. dsDNA: double-stranded deoxyribonucleic acid; ANA: antinuclear antibodies; SSA/Ro: Sjögren’s syndrome-related antigen A/Ro; SSB/La: Sjögren’s syndrome-related antigen B/La; g/dL: grams per deciliter; cells/mm^3^: cells per cubic millimeter; mm/hour: millimeters per hour; mg/L: milligrams per liter; g/L: grams per liter; IU/mL: international units per milliliter; U/mL: units per milliliter.

Laboratory parameter	Patient result	Reference value
Hemoglobin	13.8 g/dL	12.0-16.0 g/dL
Leukocytes	10,730 cells/mm^3^	4,000-11,000 cells/mm^3^
Absolute neutrophil count	6,690 cells/mm^3^	1,500-7,500 cells/mm^3^
Absolute lymphocyte count	3,150 cells/mm^3^	1,000-4,000 cells/mm^3^
Platelet count	363,000 cells/mm^3^	150,000-400,000 cells/mm^3^
Erythrocyte sedimentation rate	13 mm/hour	≤20 mm/hour
C-reactive protein	26 mg/L	<8 mg/L
Urea	0.21 g/L	0.15-0.45 g/L
Creatinine	7 mg/L	5-11 mg/L
Anti-dsDNA antibodies	31.89 IU/mL	Negative: ≤30 IU/mL; Positive: >30 IU/mL
ANA	Negative	Negative: <1:80; Positive: ≥1:80
Anti-SSA/Ro antibodies	Negative	Negative: <7 U/mL; Positive: ≥7 U/mL
Anti-SSB/La antibodies	Negative	Negative: <7 U/mL; Positive: ≥7 U/mL

**Figure 4:**
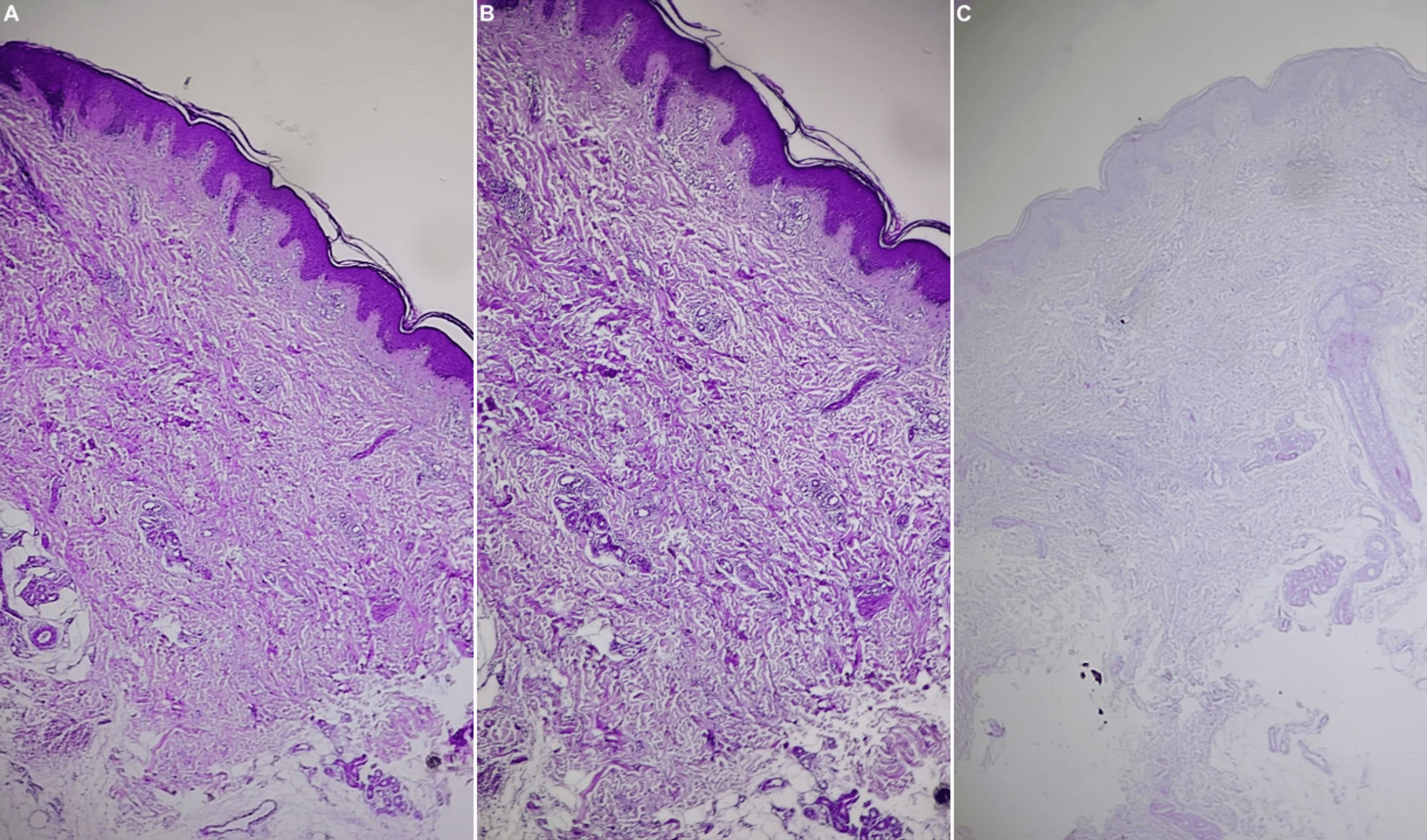
Histopathologic findings from an active lesion. (A) Mildly acanthotic epidermis without basal layer involvement, overlying a fibrotically remodeled dermis (hematoxylin-eosin-saffron (HES), ×40). (B) Dense lymphoplasmacytic inflammatory infiltrate in perivascular and periadnexal areas within the fibrotically remodeled dermis, consistent with chronic dermal inflammation (HES, ×100). (C) Absence of significant mucin deposition (Alcian blue, ×20).

Skin biopsy from an active lesion showed a mildly acanthotic epidermis without basal layer involvement. The dermis displayed fibrous remodeling and a dense lymphoplasmacytic inflammatory infiltrate in perivascular and periadnexal areas, consistent with chronic dermal inflammation. Periodic acid-Schiff staining showed no basement membrane thickening, and Alcian blue staining was negative for significant mucin deposition (Figure 4).

These findings argued against the main differential diagnoses, particularly SCLE and eczema.

In view of the patient’s rheumatic history, transthoracic echocardiography was performed and showed residual rheumatic mitral valve remodeling without visible regurgitation, left ventricular dilatation, pulmonary hypertension, or pericardial effusion, supporting chronic rheumatic valvular sequelae without evidence of active carditis or hemodynamic repercussions. Historical antistreptolysin O (ASO) titers were recorded at 360 and 226 IU/mL, supporting prior streptococcal immune exposure but not establishing contemporaneous streptococcal infection at the time of dermatologic evaluation. No recent pharyngitis or otolaryngologic infectious episode was reported; ASO and anti-DNase B titers were therefore not performed.

In the context of recurrent non-pruritic annular plaques, documented ARF history with residual valvular involvement, and exclusion of key mimickers, erythema marginatum was retained as a clinicopathologic diagnosis of exclusion [2,8-10].

The patient continued secondary prophylaxis with benzathine benzylpenicillin. No topical or systemic anti-inflammatory therapy, including corticosteroids or salicylates, was initiated because there was no evidence of active carditis or severe systemic rheumatic activity requiring such treatment.

**Figure 5:**
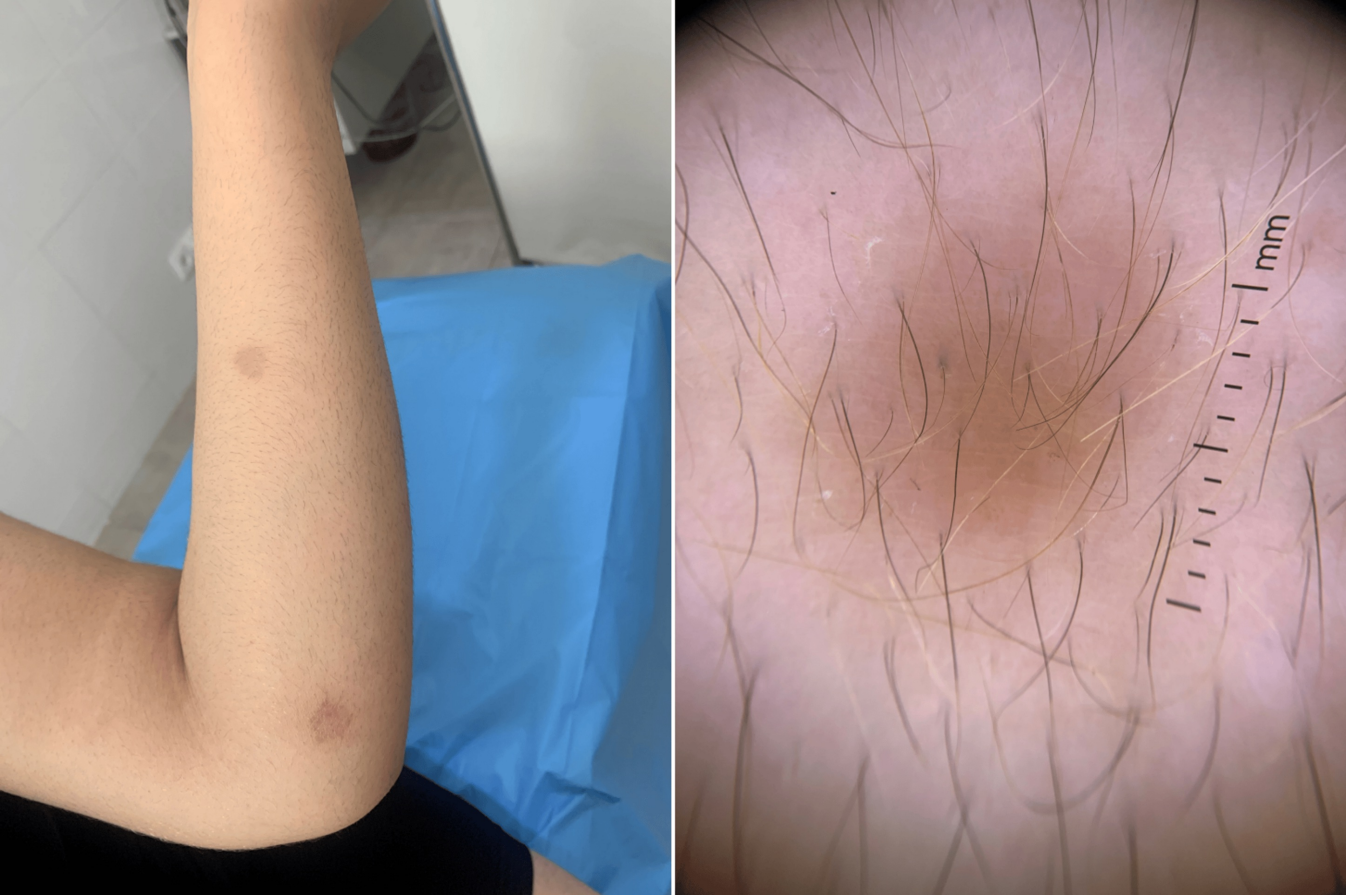
Follow-up clinical image showing regression of the plaques, with progressive flattening and decreased erythema.

At review after the histopathology results, the plaques showed progressive flattening with decreased erythema (Figure 5), with no new lesions appearing. The patient remained afebrile and clinically stable.

## Discussion

Erythema marginatum is a cutaneous manifestation of acute rheumatic fever (ARF), classified among the major Jones diagnostic criteria, although infrequently observed, reported in less than 6% of cases, with adult presentations being particularly uncommon [1,2,4-6,8].

It may precede, coincide with, or follow episodes of carditis, and has also been reported in patients with chronic carditis [2]. This point is particularly relevant in the present case, in which the patient had a documented history of ARF with residual rheumatic mitral valve involvement and recurrent annular eruptions, but no evidence of active carditis at dermatologic evaluation.

ARF is a post-streptococcal immune-mediated disease driven by cross-reactive humoral and cellular immune responses. Group A Streptococcus activates B- and T-cell responses against bacterial epitopes, and molecular mimicry with host tissues promotes immune-complex formation, inflammation, and tissue injury involving the heart, joints, skin, and central nervous system [1,11]. For the cutaneous eruption itself, the precise lesion-specific mechanism of erythema marginatum remains incompletely defined. The reported clinical and histopathologic overlap with urticaria, suggesting a shared immunologic pathway, with the shared nonspecific sparse superficial perivascular lymphocytic infiltrate, interstitial neutrophils and eosinophils, supports a nonspecific immune-mediated perivascular inflammatory pattern rather than a pathognomonic process [8-10].

Classically, erythema marginatum presents as transient, non-pruritic, annular erythematous plaques with central clearing, predominantly on the trunk and proximal limbs, usually sparing the face. The lesions are evanescent, may appear and fade within hours, and may recur during rheumatic activity [2,3]. Adult reports similarly describe recurrent annular eruptions with initial diagnostic confusion and delayed recognition [4,6]. In the present case, the two-year history of recurrent, asymptomatic annular plaques, the prior clinical suspicion of dermatophytosis and SCLE, and the underlying rheumatic history illustrate the diagnostic difficulty of recognizing erythema marginatum in adults.

The clinical overlap with other annular dermatoses is central to this case. SCLE, eczema, tinea corporis, psoriasis, erythema annulare centrifugum, urticaria, and other figurate eruptions may all enter the differential diagnosis of annular plaques [7,8]. In this setting, clinicopathologic correlation is essential. The recurrent, non-pruritic annular morphology and the rheumatic context favored erythema marginatum, whereas the absence of supportive clinical, serologic, and histopathologic findings for connective tissue disease or infection argued against the main mimickers.

To our knowledge, no specific dermoscopic pattern has been defined for erythema marginatum in the literature. In our patient, dermoscopy showed a faint erythematous background with sparse dotted vessels, corresponding to a nonspecific inflammatory pattern.

Histopathology in erythema marginatum is variable and not pathognomonic. Reported patterns include perivascular neutrophilic and mononuclear infiltrates in the papillary and upper reticular dermis, but also perivascular lymphohistiocytic inflammation without neutrophils, leukocytoclasia, vasculitis, or interface change [2,6,9,10]. Recent dermatologic literature also emphasizes the clinical and histopathologic overlap between erythema marginatum and urticaria, reinforcing that the diagnosis remains primarily clinical and contextual rather than histologically specific [8]. Therefore, biopsy is most useful not as a stand-alone confirmatory test, but as a tool to exclude mimickers and support the diagnosis when morphology and rheumatic context are concordant [2,8-10].

In this case, the absence of interface dermatitis, basal vacuolar alteration, basement membrane thickening, and dermal mucin deposition argued against cutaneous lupus erythematosus; the lack of a spongiotic dermatitis pattern argued against eczema; the absence of fungal organisms on Periodic acid-Schiff staining did not support dermatophytosis; and the lack of a psoriasiform epidermal reaction did not support psoriasis.

Overall, the chronic dermal inflammation, characterized by perivascular and periadnexal lymphoplasmacytic infiltrates within a fibrotically remodeled dermis, fell within the variable nonspecific histopathologic patterns reported in erythema marginatum and was interpreted as supportive in the appropriate clinicopathologic setting [2,8-10].

Inflammatory polyarthralgia initially contributed to the consideration of systemic inflammatory disease. However, the immunologic profile was not supportive of systemic lupus erythematosus: anti-dsDNA antibodies were only weakly positive, while ANA, anti-SSA/Ro antibodies, and anti-SSB/La antibodies were negative. With the histopathology not showing features supporting cutaneous lupus erythematosus, the isolated weak anti-dsDNA antibody positivity was therefore interpreted cautiously and was not considered sufficient to support lupus in the absence of concordant serologic and histopathologic findings.

The diagnosis retained was erythema marginatum in a patient with prior ARF and residual rheumatic valvular involvement, without evidence of active carditis or severe systemic rheumatic activity requiring anti-inflammatory therapy. Therefore, management remained conservative. The patient continued secondary prophylaxis with benzathine benzylpenicillin, and cardiac and rheumatologic follow-up were maintained. This approach is consistent with the current recommendations, where anti-inflammatory therapy with corticosteroids or salicylates is considered only in the presence of systemic rheumatic activity or active carditis [11]. In isolated cutaneous manifestations of rheumatic fever, such as erythema marginatum, specific treatment is unnecessary, as lesions typically resolve once rheumatic inflammation subsides [2,3].

This case has several strengths. First, adult erythema marginatum is rare; documentation in this age group adds valuable insight to the literature, especially with a two-year relapsing course that deviates from the reported acute, short-lived eruption [4,6]. Second, it illustrates the diagnostic challenge created by overlap with annular dermatoses, particularly SCLE, eczema, and dermatophytosis [7,8]. Third, it emphasizes the correct role of histopathology in erythema marginatum: not to confirm the diagnosis in isolation, but to exclude mimickers and support a clinicopathologic diagnosis when the clinical morphology and rheumatic context are concordant [2,8-10].

## Conclusions

This case highlights the importance of considering erythema marginatum in adults with recurrent annular eruptions and a history of acute rheumatic fever. Because erythema marginatum lacks pathognomonic histopathologic features and may mimic subacute cutaneous lupus erythematosus, eczema, or dermatophytosis, diagnosis requires careful clinicopathologic correlation and exclusion of mimickers. Early recognition prevents misdiagnosis and inappropriate treatment, while ensuring appropriate rheumatic and cardiac follow-up.
